# Toll-like receptor 1 polymorphism is associated with impaired immune tolerance, dysregulated inflammatory responses to *Borrelia burgdorferi*, and heightened risk of post-infectious Lyme arthritis

**DOI:** 10.3389/fimmu.2025.1711765

**Published:** 2025-11-17

**Authors:** Morgan A. Williams, Sergio A. Hernandez, Sheila L. Arvikar, Katherine B. Sulka, Franc Strle, Christopher C. Wells, Tanja Petnicki-Ocwieja, Allen C. Steere, Klemen Strle

**Affiliations:** 1 Department of Molecular Biology and Microbiology, Tufts University School of Medicine, Boston, MA, United States; 2 Division of Rheumatology, Allergy and Immunology, Massachusetts General Hospital, Harvard Medical School, Boston, MA, United States; 3 Department of Infectious Diseases, University Medical Centre Ljubljana, Ljubljana, Slovenia; 4 Department of Immunology, Tufts University Graduate School of Biomedical Sciences, Boston, MA, United States

**Keywords:** Lyme disease, *Borrelia burgdorferi*, toll-like receptors, innate immune tolerance, inflammation, Lyme arthritis, innate immunology

## Abstract

**Introduction:**

Clinical presentation of Lyme disease is largely due to host immune response to infection. Previously, we identified a variant (1805GG) in the TLR1 gene, a key immune sensor for *Borrelia burgdorferi*, which was associated with excessive inflammation and severe disease. Herein we examined the mechanism by which this variant leads to dysregulated immunity.

**Methods:**

We found that patients with post-infectious Lyme arthritis, a condition characterized by marked persistent synovitis in joints, have a higher frequency of TLR1-1805GG compared to those whose arthritis resolves with antibiotics. To explore the possibility that this genotype-phenotype association was due to excessive inflammation, we then tested the functional impact of TLR1-1805GG on inflammatory responses and immune tolerance in PBMCs with or without this SNP and in THP-1 cell lines lacking TLR1.

**Results:**

In response to *B. burgdorferi* stimulation, PBMCs with TLR1-1805GG had greater transcriptional upregulation of ~1200 immune-related genes and significantly higher cytokine levels in supernatants compared to cells without this variant. Moreover, repeat *B. burgdorferi* stimulation, which mimics tolerogenic conditions during the infection, failed to induce innate immune tolerance in PBMCs with TLR1-1805GG, or in THP-1 cells lacking TLR1, resulting in seemingly unabated immune activation consistent with marked inflammation in Lyme arthritis joints.

**Conclusions:**

These results suggest that excessive inflammation in patients with TLR1-1805GG variant appears to be due to immune dysregulation and inability to induce immune tolerance. The findings help explain how early events during the infection may contribute to sustained immune activation after antibiotics and point to the role of TLR1 signaling in immune regulation.

## Introduction

Lyme disease, caused by the tick-borne spirochetes of the *Borrelia burgdorferi* sensu lato (s.l.) complex, is the most common vector-borne disease in North America and Europe. An expanding erythema migrans (EM) skin lesion is typically the first sign of the disease. In untreated individuals, spirochetes may disseminate to other organ systems, namely the central nervous system, heart, or joints (1, 2). In North America, *Borrelia burgdorferi* is the major causative agent of Lyme disease, while in Europe the disease is mainly caused by *Borrelia afzelii* and *Borrelia garinii*.

Lyme arthritis (LA) is the most common extra-cutaneous manifestation of Lyme disease in the United States (2). As with other manifestations of Lyme disease, patients with LA usually respond to antibiotic therapy and their arthritis resolves (antibiotic-responsive LA). However, a small subset of patients develop marked persistent synovitis despite 2–3 months of treatment with oral and/or intravenous (IV) antibiotics (post-infectious LA) (3, 4). With little evidence to suggest a persistent *Borrelia* infection in these patients after antibiotic treatment, this clinical outcome indicates a post-infectious immune-mediated process (4, 5).

The immunopathogenesis of Lyme disease involves a complex interplay between *Borrelia* and the host. The innate immune system serves as the first line of defense by recognizing a multitude of *B. burgdorferi* s.l. lipoproteins (6) via Toll-Like Receptors (TLRs) 1 and 2 (7), flagellar proteins via TLR5 (8), and nucleic acids via TLR7, 8, and 9 (9–11). Activation of these pathways triggers a robust production of inflammatory cytokines and chemokines, resulting in recruitment and activation of immune cells to sites of infection (12–16).

Although a robust immune response is important in controlling the infection, excessive and prolonged inflammation is associated with more severe disease. For example, in patients with EM, high levels of IL-6, IFNγ, and IFN-inducible chemokines CXCL9 and CXCL10 are associated with greater number of symptoms in early infection. Similarly, exceptionally high levels of these mediators in joints are associated with immune dysregulation, marked immune cell infiltrates, and persistent synovitis in patients with post-infectious LA (14, 17–19). Sustained elevated immune responses in patients with post-infectious LA suggest that the usual mechanisms to limit tissue-damaging inflammation, such as immune tolerance, may be broken – a phenomenon associated with autoimmune diseases.

Host genetic variation in immune-related genes is thought to play a significant role in shaping clinical outcomes in Lyme disease (17, 20–24). Previously, we found that a single nucleotide polymorphism (SNP) in TLR1, a key *Borrelia*-sensing receptor, is associated with excessive inflammation, greater number of symptoms in EM patients, and increased prevalence of post-infectious LA (17). This SNP results in a thymine-to-guanine transversion at position 1805 in TLR1, leading to an isoleucine-to-serine substitution (13, 25) within a short 6 amino acid sequence in the cytoplasmic region which is responsible for TLR1 cell surface trafficking. The homozygous variant of this SNP (TLR1-1805GG) results in decreased TLR1 translocation to the cell surface, impaired downstream signaling, and diminished inflammatory responses (17, 26). Surprisingly, in Lyme disease, this SNP is associated with exacerbated inflammatory responses to *B. burgdorferi* and more severe disease, presumably due to a loss of appropriate regulation (17, 27).

In this study, we evaluate the risk-association between the TLR1-1805G SNP and post-infectious LA in an expanded cohort of patients and demonstrate that excessive inflammation associated with this SNP may be due to a break in innate immune tolerance. We reveal a central modulatory role of TLR1 in immune responses to *Borrelia*. Moreover, the findings underscore the importance of host genetic variation in immune responses to infection and long-term clinical outcomes of disease.

## Methods

### Study patients

Pooled analysis of data from two studies involved 372 patients with Lyme disease: 285 with LA and 87 with EM. Patients with LA were seen by Drs. Allen Steere and Sheila Arvikar at Tufts Medical Center or Massachusetts General Hospital Rheumatology clinics over a 30-year period between 1987 and 2017. Of the 285 LA patients, 114 had antibiotic-responsive LA, and 171 had post-infectious LA (3, 28, 29). The LA cohort included 138 patients from a previous cohort (17) and 147 new patients. The high proportion of patients with post-infectious LA is reflective of the clinic serving as a referral center. Patients with EM were seen at the Lyme borreliosis outpatient clinic at the University Medical Centre Ljubljana in Slovenia. All patients met the Centers for Disease Control and Prevention (CDC) diagnostic criteria for Lyme disease.

### TLR1–1805 genotyping

Genotyping was performed using PCR amplification followed by restriction fragment length polymorphism (RFLP) as described previously (17). Briefly, total genomic DNA was isolated from blood or PBMCs using QIAamp DNA Blood Mini Kit (Qiagen). DNA was amplified in 25µL reactions: DNA ≥ 50ng, 2X DreamTaq PCR Master Mix (Thermo Fisher Scientific), and 0.4µM forward [GCAGGGGACAATCCATTCCAA] and reverse primers [CCCAGAAAGAATCGTGCCCA] (IDT). PCR products were digested with PstI (New England Biolabs) for 1 hour at 37°C followed by electrophoresis. Wild-type (WT) THP-1 cells (ATCC) were confirmed to carry the reference TLR1-1805TT genotype.

General population TLR1–1805 genotype frequency was ascertained from the 1000 Genomes Project (rs5743618). Non-European (non-EUR) ancestry includes individuals from Africa, South and Central America, and East and South Asia. European ancestry (EUR) includes individuals from Britain, Finland, Spain, Tuscany, and the U.S. (30).

### Preparation of *B. burgdorferi*

Low-passage (<3) *B. burgdorferi* isolates (RST1, OspC type A) from a U.S. patient with EM (17, 31) and *B. afzelii*, *B. garinii*, and *B. burgdorferi* from Slovenian patients with EM, were cultured to mid-log phase in complete Barbour-Stoenner-Kelly (BSK) medium containing 6% rabbit serum (31). Spirochetes were quantified by optical density (OD 600) and resuspended in cell-culture medium (RPMI 1640, Gibco) containing 10% heat-inactivated fetal bovine serum (HI-FBS; Gibco) (17, 31, 32).

### Cell culture

PBMCs were isolated from buffy coats obtained from healthy human donors (NY Blood Center) as previously described (17, 31). While donors had no known history of Lyme disease and no active infection (within 30 days of blood collection), prior exposure to *Borrelia* could not be fully excluded. For experiments, PBMCs were washed, enumerated, and seeded at 2x10^5^/well in 96-well tissue-culture plates (Corning) and rested overnight at 37°C, 5% CO_2_ prior to stimulation with *Borrelia* at a multiplicity of infection (MOI) of 20. WT THP-1 cells, TLR1-Knockout (KO) THP-1 cells (Ubigene), and TLR2-KO THP-1 cells (InvivoGen) were grown to ~80% confluency in 96-well tissue culture plates (2x10^5^/well) at 37°C, 5% CO_2_ in cell-culture medium, then differentiated into macrophages using 200nM phorbol 12-myristate 13-acetate (PMA; Sigma-Aldrich). Adherent THP-1 macrophages were washed and rested for 24hr in cell culture medium prior to stimulation with *Borrelia*.

### Tolerance experiments

PBMCs or THP-1 macrophages were co-cultured with *B. burgdorferi* or Pam_3_CSK_4_ (300 ng/ml; InvivoGen) for 24hr (Stimulated). The 300 ng/mL Pam_3_CSK_4_ dose is a commonly used dose for TLR1/2 stimulation (33, 34). Cells were washed, rested in fresh cell-culture medium for 1hr at 37°C, 5% CO_2_, then restimulated with *B. burgdorferi* or Pam_3_CSK_4_ (Restimulated). Supernatants were collected at 24hr after stimulation and again at 24, 48, and 96hr after restimulation for cytokine analysis. IL-6 was selected as the primary readout for tolerance experiments because it is a canonical marker of innate immune activation and tolerance, reliably reflects the magnitude of inflammatory activation, and serves as a central inflammatory mediator in joint pathogenesis, including in patients with Lyme arthritis (14, 31, 35–38). RNA sequencing was performed on cells harvested 16hr after stimulation and 6hr after restimulation.

### Cytokine and chemokine determinations

Levels of mediators (GM-CSF, IFNγ, CXCL9, CXCL13, IFNα, TNF, CCL4, CCL5, IL-6, CCL3, IL-1β, CCL2, Fractalkine, CXCL10, IL-17A, G-CSF, IL-8, M-CSF, IL-10, IL-12p70, and IL-23) were assessed from supernatants (1:25 dilution) using bead-based multiplex assays (EMD Millipore). Data were acquired using a Luminex^®^ 200 Instrument (Luminex) and Xponent v.4.3.309.1 software. IL-6 levels were also assessed (1:10 dilution) using IL-6 BD OptEIA™ ELISA kits (BD Biosciences) according to manufacturer’s instructions. Data were collected and analyzed using BioTek Gen5 software (Agilent).

### Transcriptomic analysis

PBMCs from one TLR1-1805TT and one TLR1-1805GG donor were stimulated with *B. burgdorferi* in three independent experiments. Following stimulation (16hr) and restimulation (6hr), PBMCs were harvested, and RNA was extracted using the RNeasy^®^ Plus Micro Kit (Qiagen). RNA-seq libraries were prepared using NEBNext Ultra-II Directional RNA Library Prep Kit (Illumina) and sequenced (150bp paired-end) using the Illumina HiSeq, generating ~25 million reads per sample by the AGTC Genomic and Bioinformatics Core Facility at Wadsworth Center, NYS Department of Health. Sequencing data were aligned to the human genome (hg38, STAR RNA-seq aligner). Omics Playground and Metascape were used for visualization and pathway analysis of DEGs (39, 40).

### Statistical analysis

GraphPad Prism-v9.5.1 and R-v4.2.3 were used for statistical analyses. TLR1-1805GG frequencies and odds ratios (OR) were compared across patient groups by Fisher’s exact tests. Logistic regression was applied across five standard genetic models with post-infectious LA as the outcome of interest. For each model, an unadjusted binomial generalized linear model (GLM) in R was used to compare the OR, 95% confidence intervals (CI), p-value, and Akaike Information Criterion (AIC). Immune mediators and RNA-seq normalized transcript counts (TPM) were compared using Welch’s t-tests. Mann-Whitney U rank-sum tests were used for groups without normal distribution. Partial Least Squares Discriminant Analysis (PLS-DA) and Variable Importance in Projection (VIP) analyses were performed using the R package “mixOmics”. IL-6 levels in tolerance experiments were compared using ANOVA followed by Tukey’s *post-hoc* test. For all figures, * P<0.05, ** P<0.01, *** P<0.001, and **** P<0.0001.

## Results

### TLR1–1805 genotype frequency in patients with Lyme disease and the general population

**Figure 1:**
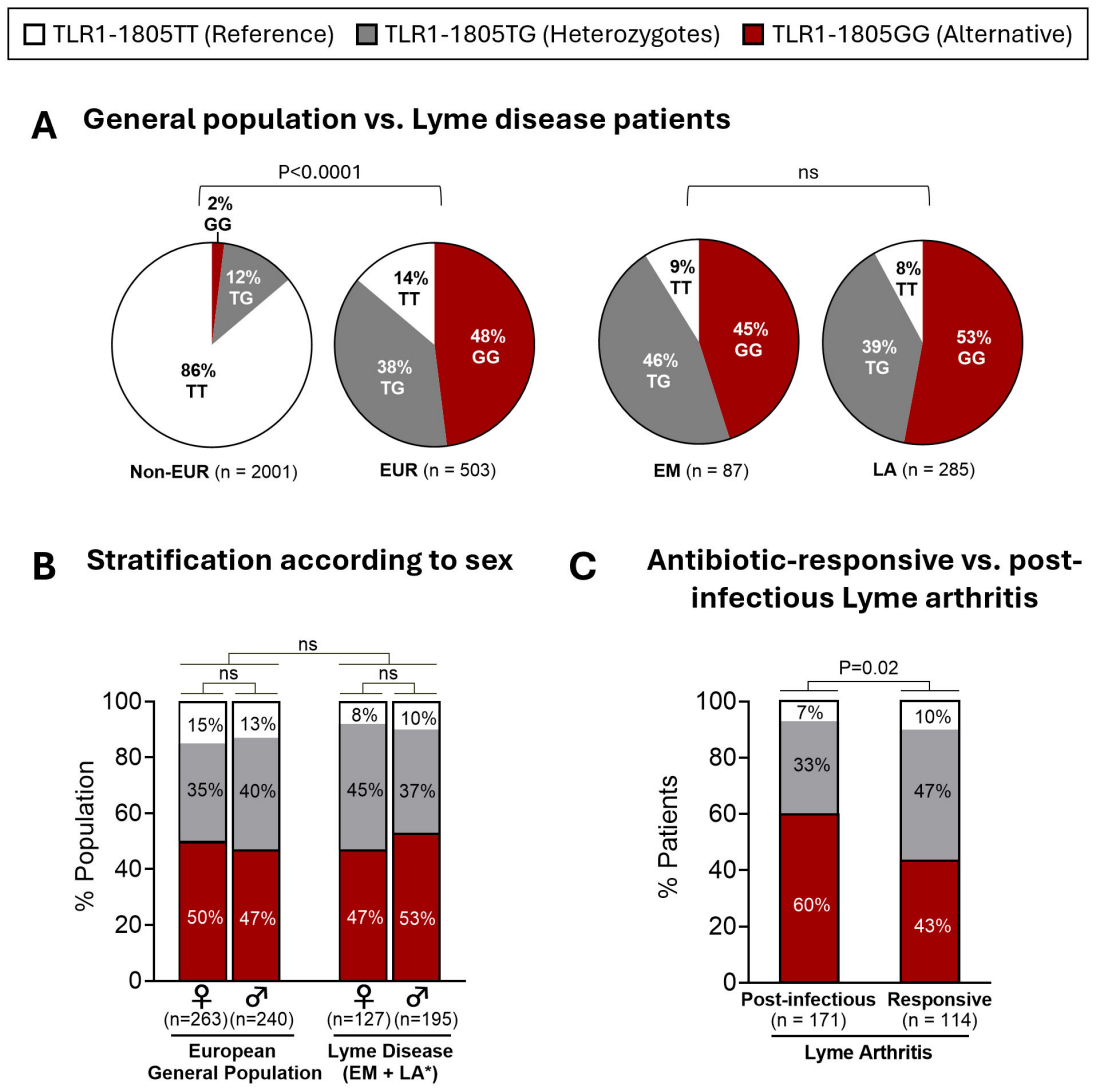
TLR1-1805 genotype frequency in patients with Lyme disease and the general population. **(A)** Distribution of TLR1-1805 genotypes in 2,504 healthy subjects [2,001 non-European descent (Non-EUR); 503 European descent (EUR)] and 372 patients with Lyme disease [87 Erythema Migrans (EM) patients; 285 patients with Lyme arthritis (LA)]. **(B)** Distribution of TLR1-1805 genotypes across biological sex in European general population [503 (263 females; 240 males)] and Lyme disease patients [322 (127 females; 195 males)] *Sex unknown for 50 LA patients. **(C)** TLR1-1805 genotype distribution of patients with LA stratified by resolution with antibiotic therapy [Responsive, n=114] or persistent arthritis [Post-infectious, n=171]. **(A–C)** Fisher’s exact tests were used to compare frequencies across general and patient population groups as well as antibiotic-responsive and post-infectious LA patient groups.

In our initial investigation of TLR1, TLR2, and TLR5 variants in Lyme disease, we found that patients with post-infectious LA had a greater frequency of TLR1-1805GG (62%) compared to patients with antibiotic-responsive LA (47%) (17). Here, we expand these findings by evaluating the TLR1–1805 genotype frequency in the general population from multiple ancestries, in a larger cohort of patients with LA, and in European patients with EM. Data from the general population were derived from the 1000 Genomes Project (30). As most of our patients with Lyme disease are of European descent, we stratified the general population into individuals of non-European (n=2001) and European descent (n=503). Consistent with previous studies (17, 25), approximately half (48%) of European individuals have both copies of the TLR1-1805G SNP (1805GG) compared to only 2% of non-European individuals (OR: 41.9; P<0.0001) (**Figure 1A**). Next, genotype frequency was assessed in 372 patients with Lyme disease, including 285 patients with LA and 87 patients with EM, a majority of whom were of European descent. Similar to the general European population, 45% of patients with EM and 53% of patients with LA carried the 1805GG genotype (**Figure 1A**). Of interest, the frequency of TLR1-1805GG in European patients with EM in this study, the majority of whom are infected with *B. afzelii*, was similar to that in U.S. patients with EM in our previous study (51%) (17), the majority of whom were infected with *B. burgdorferi*, suggesting that the *Borrelia* species alone were not the major contributor to differences in TLR1-1805GG frequency among these cohorts. Likewise, the GG frequency in LA patients was similar in both studies (TLR1-1805GG was present in 53% of LA patients in the current study and in 55% in our previous study). No sex-dependent differences in the TLR1–1805 genotype frequencies were observed (**Figure 1B**).

Next, we compared TLR1–1805 genotype frequencies in patients with antibiotic-responsive LA versus those with post-infectious LA. The 1805GG genotype was significantly more frequent in patients with post-infectious LA (60%) than in those with antibiotic-responsive LA (43%) (OR: 2; P = 0.02) (**Figure 1C**). Genetic modeling of TLR1–1805 further supported an association between a G-allele recessive model and clinical outcomes of LA (OR: 2.01, 95% CI: 1.24-3.25, P = 0.004) (**Supplementary Table 1**). Although there was a trend toward a higher frequency of the TLR1-1805GG variant in LA patients compared to EM patients and the general European population, these differences were not significant and appear to be driven primarily by patients with post-infectious LA. Similar results were also observed in our initial study (17). Collectively, these data suggest that the TLR1-1805GG variant is associated with excessive immune responses and, as a result, greater disease severity in Lyme arthritis, rather than with overall susceptibility to Lyme disease.

### TLR1-1805GG is associated with greater inflammatory responses to *B. burgdorferi*

**Figure 2:**
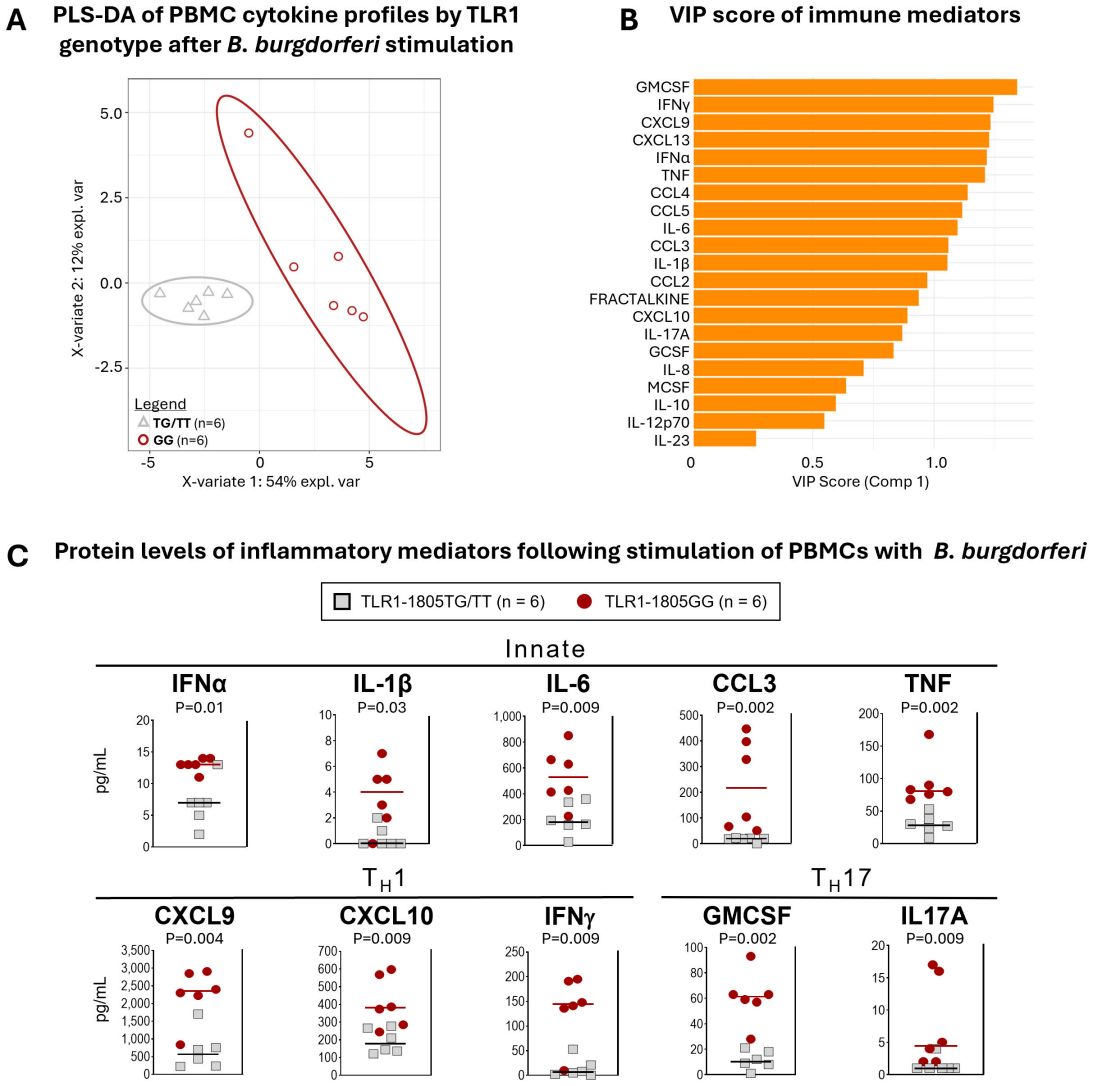
TLR1-1805GG is associated with greater inflammatory responses to *B. burgdorferi*. **(A)** Partial least squares discriminant analysis (PLS-DA) of cytokine expression profiles in PBMCs stimulated with *B. burgdorferi*, grouped by TLR1-1805 genotype [TG/TT = gray triangles; GG = red circles]. Each point represents one donor. Ellipses indicate 95 % confidence regions for each genotype group, and the percent variance explained by each component (X-variate 1 and 2) is shown on the axes. **(B)** Variable importance in projection (VIP) scores for immune mediators contributing to separation of TLR1-1805GG and TG/TT groups in the PLS-DA model. Higher VIP scores indicate greater influence on group discrimination. **(C)** Protein levels of inflammatory mediators in PBMC supernatants following stimulation with *B. burgdorferi*: TLR1-1805TG/TT = grey squares, n=6; TLR1-1805GG = red circles, n=6. Lines represent median values. Statistical comparisons between genotypes were performed using Mann-Whitney U test; p-values are indicated.

Previously, we demonstrated that Lyme disease patients with both copies of TLR1-1805G SNP had significantly higher levels of inflammatory mediators, including IL-6, CXCL9, and CXCL10, in serum or joint fluid compared to those with only one copy (1805TG) or those with ancestral/reference alleles (1805TT) (17). These results generated the link between TLR1-1805GG, elevated immune responses, and more severe disease. Here, we tested the possibility that this SNP contributes to excessive inflammatory responses to *B. burgdorferi* in *ex vivo* PBMC cultures. PBMCs from six donors with the 1805GG genotype and six donors with 1805TG/TT genotypes were stimulated with *B. burgdorferi* RST1 strain (17) for 24hr and 21 cytokines and chemokines were quantified in cell supernatants. PLS-DA incorporating all mediators revealed complete discrimination between 1805GG and 1805TG/TT genotypes (**Figure 2A**). VIP scores ordered the mediators that served as key discriminators/differentiators between the two groups (**Figure 2B**). Ten immune mediators associated with innate (IFNα, IL-1β, IL-6, CCL3, TNF), Th1 (CXCL9, CXCL10, IFNγ), or Th17 (GM-CSF, IL-17A) responses were significantly elevated (P <0.01) in PBMCs with 1805GG compared to 1805TG/TT (**Figure 2C**). These ten mediators were selected from a larger panel of 21 analytes (**Figure 2B**) because they are representative of both innate and adaptive Th1 and Th17 immune responses. Collectively, these results indicate that the TLR1-1805GG genotype increases activation of a broad range of innate and adaptive inflammatory responses to *B. burgdorferi*, consistent with observations in patients and animal models (17, 27, 36).

### TLR1–1805 SNP is associated with a break in innate immune tolerance and sustained inflammation

**Figure 3:**
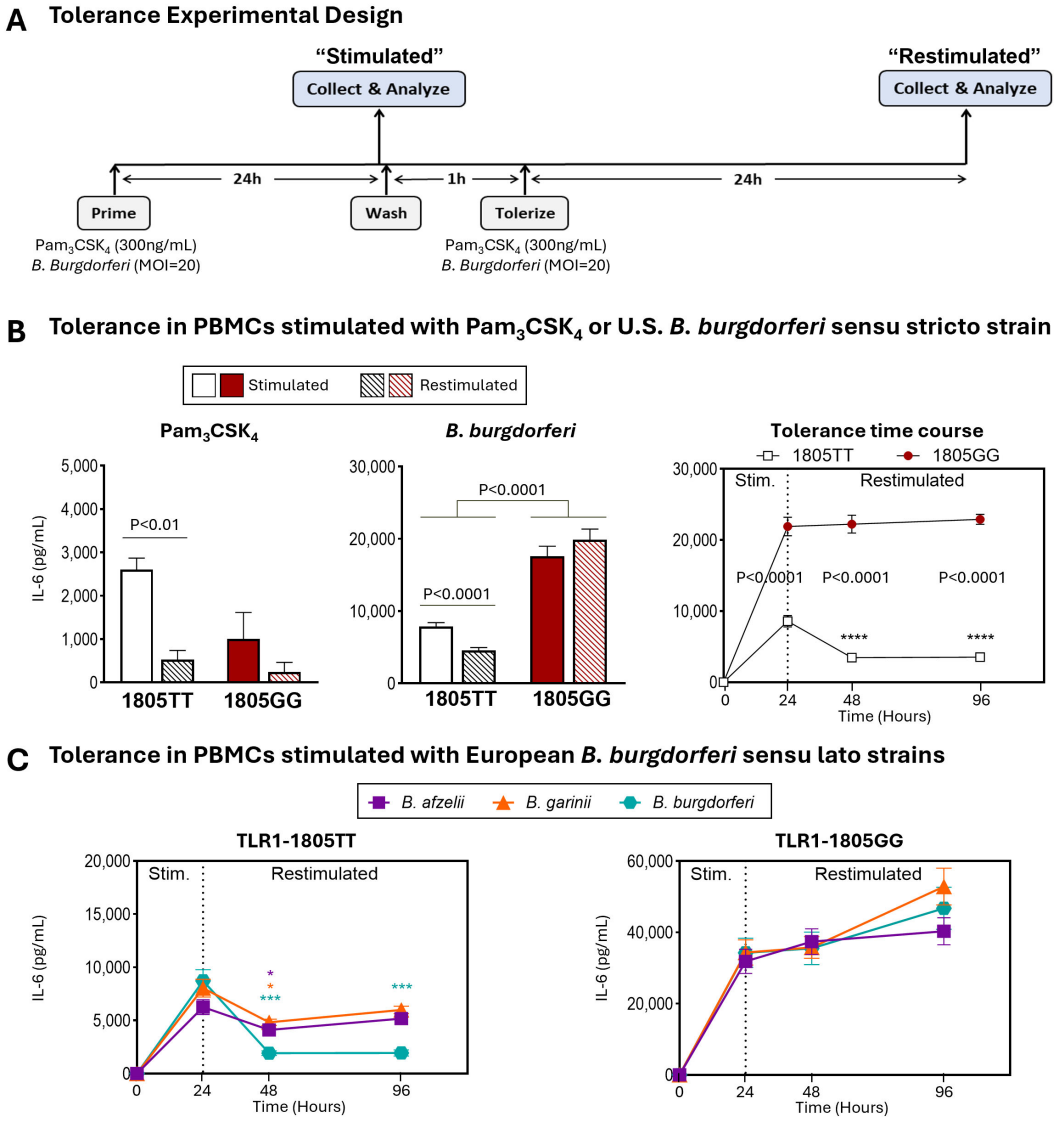
TLR1-1805 SNP is associated with a break in innate immune tolerance and sustained inflammation. **(A)** Schematic of experimental design for induction of innate immune tolerance. **(B)** IL-6 protein levels in PBMCs from healthy donors carrying TLR1-1805TT [white bars, n=3] or TLR1-1805GG [red bars, n=3] stimulated for 24hr with Pam3CSK4 (left panel) or *B. burgdorferi* (middle panel). Cells were then washed, rested, and restimulated for an additional 24hr [striped bars]. Statistical significance was determined by Welch’s t-test. IL-6 production kinetics following restimulation at 24hr post-primary stimulation are shown in the right panel [white squares=TT; red circles=GG]. Mann-Whitney U tests were used to assess differences between genotypes and time points. **(C)** Protein levels of IL-6 in PBMCs with TLR1-1805TT (left panel) or TLR1-1805GG (right panel) stimulated and restimulated with European *B. burgdorferi* sensu lato strains: *B. afzelii* [purple squares], *B. garinii* [orange triangles], and *B. burgdorferi* [teal hexagons]. Mann-Whitney U tests compared stimulated (Stim.) versus restimulated time points. **(B-C)** Asterisks (*) indicate comparisons between stimulated and restimulated conditions; written p-values indicate genotype comparisons. Error bars denote mean ± SEM. ****P<0.0001, ***P<0.001, **P<0.01, *P<0.05.

Excessive inflammation observed with TLR1-1805GG alleles could result from a failure of immune tolerance to continued stimulation with *B. burgdorferi* which may occur during the course of natural infection. To test this possibility, we assessed the ability of TLR1-1805GG PBMCs to induce tolerance following repeated stimulation with *B. burgdorferi* by measuring IL-6 production (41, 42). PBMCs from three donors with the 1805GG genotype and three donors with the 1805TT genotype were stimulated with the TLR1/2 agonist Pam_3_CSK_4_ or *B. burgdorferi* for 24hr, rested, and restimulated for another 24hr (**Figure 3A**). The PBMC with TLR1 1805TT demonstrated a significant reduction in IL-6 protein levels (P<0.0001) after restimulation with *B. burgdorferi*, presumably due to induction of tolerance which limits inflammatory responses to repeated pathogen stimulation (**Figure 3B**, white bars). In contrast, 1805GG PBMCs induced greater IL-6 levels after primary stimulation with *B. burgdorferi* (**Figure 3B**, red bars) compared to 1805TT (~18,000 pg/mL vs 8,000 pg/ml; p<0.0001), and these levels remained elevated after restimulation (**Figure 3B**, right panel) which suggests these cells are unable to induce tolerance. Similar findings were observed with all three pathogenic European *Borrelia* s.l. species (**Figure 3C**), suggesting this phenotype is not *Borrelia* species-specific. Stimulation with Pam_3_CSK_4_, a specific TLR1/2 agonist, did not result in greater inflammatory responses in 1805GG PBMCs compared to 1805TT (**Figure 3B**, left panel), consistent with previous observations (17). Collectively, these results suggest that the TLR1–1805 SNP, which results in loss of TLR1 on cell surface, leads to a break in immune tolerance to *B. burgdorferi* and unabated inflammation, reminiscent of findings in synovial fluid of patients with post-infectious LA (17).

### TLR1 modulates immune responses to *B. burgdorferi*

**Figure 4:**
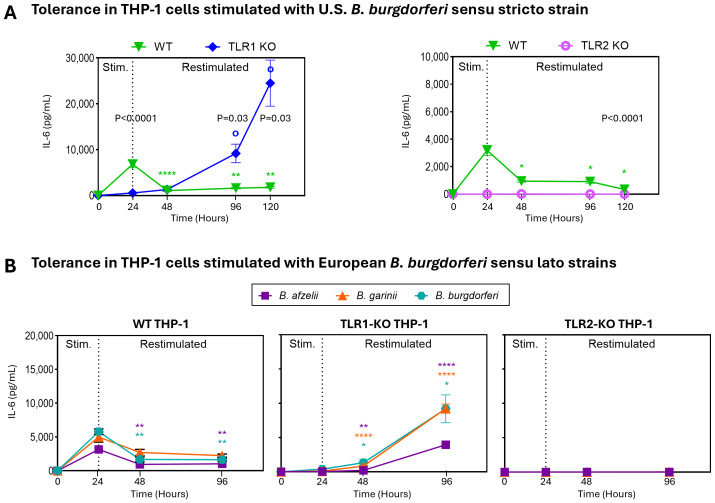
TLR1 modulates immune responses to *B. burgdorferi*. **(A)** IL-6 levels in wild-type [WT; green inverted triangles], TLR1 knockout [TLR1-KO; blue diamonds], and TLR2 knockout [TLR2-KO; pink tori] THP-1 cells stimulated with *B. burgdorferi* for 24hr, then restimulated. Levels of IL-6 were assessed at baseline, 24h (primary stimulation), and at 48h, and 96h and 120hr (post restimulation). Statistical significance was determined using Mann-Whitney U tests. Open circles (o) indicate comparisons of restimulated time points to the 24hr primary stimulation in TLR1-KO THP-1s; asterisks (*) indicate corresponding comparisons in WT THP-1s; written p-values indicate comparisons between WT and KO THP-1s. **(B)** IL-6 levels in WT (left panel), TLR1-KO (middle panel), and TLR2-KO (right panel) supernatants after stimulation and restimulation with European *B. burgdorferi* sensu lato strains: *B. afzelii* [purple squares], *B. garinii* [orange triangles], and *B. burgdorferi* [teal hexagons]. Statistical significance between stimulated and restimulated conditions was determined by Welch’s t-test. **(A, B)** Error bars represent mean ± SEM. ****P<0.0001, **P<0.01. * or o P<0.05.

To directly assess the impact of TLR1 signaling on tolerance to *B. burgdorferi*, we tested THP-1 macrophages with (WT) or without TLR1 (TLR1-KO). Similar to our results in 1805TT PBMCs, WT THP-1 cells induced tolerance upon restimulation with *B. burgdorferi* leading to significant reduction in IL-6 production (P<0.0001) that was sustained for 120hr (**Figure 4A**). In contrast, IL-6 levels in TLR1-KO cells continued to increase after restimulation and reached significantly higher concentrations at 96hr post-stimulation compared to WT cells (P<0.03). Notably, at 120hr post stimulation, IL-6 levels reached ~25,000pg/ml in TLR1-KO compared to ~2000pg/ml in WT cells (P<0.03) (**Figure 4A**). IL-6 levels in TLR2-KO macrophages were at or below the limit of detection at all timepoints (**Figure 4A**). As with PBMCs, similar results were observed with all three pathogenic European *B. burgdorferi* s.l. species (**Figure 4B**). These data suggest that whereas TLR2 is critically important for inflammatory responses to *B. burgdorferi* (8), functional cell surface TLR1 signaling plays an important regulatory role. Signaling via TLR1 is important in the induction of tolerance and thus in limiting host immune responses to *B. burgdorferi* during repeated stimulations.

### Transcriptomic assessment of immune tolerance in TLR1-1805GG PBMC

**Figure 5:**
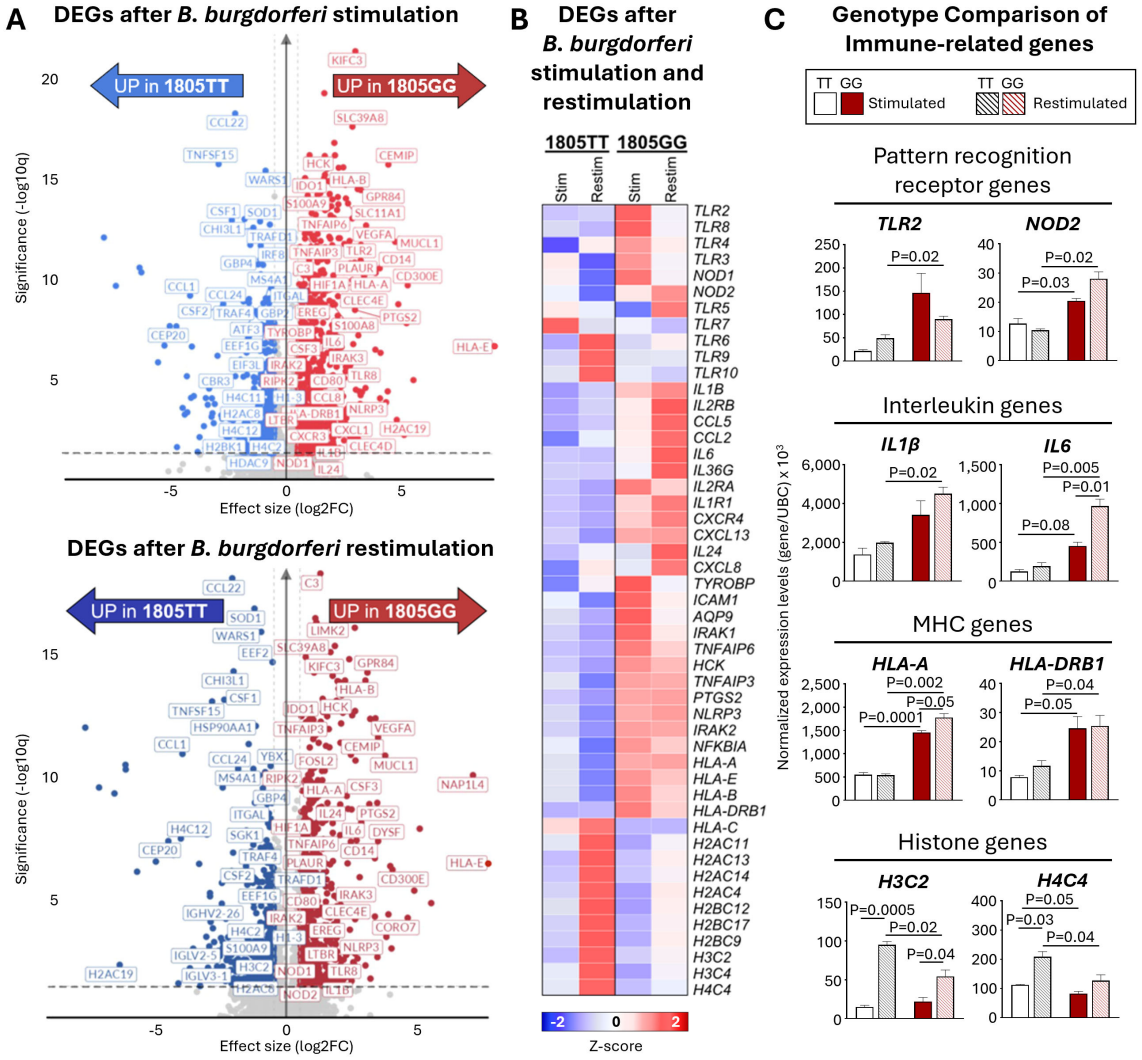
Transcriptomic assessment of immune tolerance in TLR1-1805GG PBMCs. **(A)** Volcano plots of significantly upregulated differentially expressed genes (DEGs) in TLR1-1805TT [blue] and TLR1-1805GG [red] donor PBMCs following *B. burgdorferi* stimulation for 16hr (top panel) and 6hr after restimulation (bottom panel). **(B)** Heatmap of selected genes illustrating expression profiles of pattern recognition receptor (PRR), interleukin (IL), major histocompatibility complex (MHC), and histone genes in PBMCs stimulated (16hr) and restimulated (6hr) with *B. burgdorferi*. **(C)** Bar graphs showing normalized gene expression (relative to *UBC*) for representative genes within each category: PRR – Toll-like receptor 2 (*TLR2*), nucleotide-binding oligomerization domain 2 (*NOD2*); Interleukin – IL1β, IL6; MHC – human leukocyte antigen A (*HLA-A*), human leukocyte antigen DRB1 (*HLA-DRB1*); Histone genes – H3 clustered histone 2 (*H3C2*), H4 clustered histone 4 (*H4C4*). TLR1-1805TT donors are shown as white bars (n=1, three technical replicates) and TLR1-1805GG donors as red bars (n=1, three technical replicates). Statistical significance was assessed by Welch’s t-test between conditions and donors. Error bars represent ± SEM from three experiments. P-values are written in graphs.

Next, we used RNA-sequencing to elucidate the range of molecular pathways that may be altered by this SNP and gain insights into their potential role in tolerance. In these experiments, PBMCs from two donors, one GG and one TT, were stimulated for 16hr with *B. burgdorferi*, rested, and then restimulated for 6hr. Compared to unstimulated cells, ~2000 genes were differentially expressed (DEGs) upon *B. burgdorferi* stimulation, (1987 in TT and 2242 in GG; Log2-fold change >0.58 or <-0.58, p-adjusted ≤0.05). Immune genes upregulated in the 1805GG PBMCs after stimulation were involved in pathogen sensing (e.g., *TLR2*, *TLR8*, *NOD1*), interleukin signaling (*IL6*, *IL1B, IL24)*, and antigen presentation *(HLA-B, HLA-DRB1)* (**Figure 5A**). In comparison, genes upregulated in cells with 1805TT were primarily associated with histone modification (**Figure 5A**). Similar DEG patterns were visualized in restimulated cells, highlighting the overall inflammatory gene profiles in TLR1-1805GG genotype (**Figure 5A**). When comparing genes across all conditions, immune genes, such as pattern recognition receptor (PRR), interleukin, or MHC genes were upregulated in both TLR1-1805GG conditions, and their expression was lower or downregulated in 1805TT conditions (**Figures 5B, C**). In contrast, many upregulated genes in 1805TT PBMCs upon restimulation were related to DNA repair, cell cycle, and chromatin organization (*H4C12*, *H3C2*, and *H4C4)* which may contribute to transcriptional regulation and induction of immune tolerance (**Figures 5B, C**). Collectively, these *in vitro* findings demonstrate that the TLR1-1805G SNP is associated with excessive immune activation in response to increased *B. burgdorferi* stimulation, including genes involved in pathogen sensing, antigen presentation, and inflammation, consistent with the greater inflammatory response and more severe disease in patients. Moreover, the data suggest that functional cell surface TLR1 signaling plays an important role in modulating the inflammatory response to *B. burgdorferi*.

## Discussion

Host genetic variation shapes the repertoire of immune responses to microbial pathogens and is considered the major determinant of disease susceptibility (43). The association of the TLR1-1805GG genotype with excessive inflammation and post-infectious LA provides an example of this concept in Lyme disease. Genes involved in innate immunity, such as *TLR1* and *TLR6*, have evolved under stronger selective pressures compared to other protein coding genes, likely due to their importance in protection from life-threatening diseases (44). Moreover, regional variations have been observed in these susceptibility loci, likely reflecting the differences in pathogen exposure. For example, variants in the TLR6-TLR1-TLR10 cluster appear to have been under strong positive selective pressure in Europeans (44) which is also consistent with the higher prevalence of the TLR1-1805G SNP in those with European ancestry as demonstrated here and in previous publications (17, 25).

In our initial study (17), we found that patients with both copies of this SNP (TLR1-1805GG) had excessive inflammatory responses in serum and joint fluid which were associated with more symptomatic early infection (EM duration: 1–2 weeks) and greater likelihood of developing post-infectious LA (arthritis duration: months-years). In the current study, we explored the potential mechanism through which this TLR1 variant may contribute to aberrant inflammation. We found that in response to repeat *B. burgdorferi* stimulation, the TLR1-1805G SNP is associated with heightened levels of key innate and adaptive Th1 and Th17 cytokines that remained elevated for days, indicative of a sustained dysregulated immune response. In contrast, cells without this SNP exhibited abrogated inflammatory responses to repeat stimulation with *B. burgdorferi*, suggestive of an appropriately regulated tolerogenic response. The inability to induce innate immune tolerance in TLR1-1805GG PBMCs was mimicked in human THP-1 macrophage cell lines lacking the TLR1 receptor, suggesting that intact TLR1 signaling likely serves an important role in modulating immune responses to *B. burgdorferi* and possibly other pathogens. The net result of disrupted TLR1 signaling in both cell models is a seemingly unabated inflammatory response consistent with sustained immune activation observed in patients with post-infectious LA.

The TLR1-1805G SNP was first described by Hawn et al. (25). Among the 17 TLR1 SNPs identified, 1805G was notable for its unique location near the transmembrane domain; a highly constrained region likely to impact function. PBMCs with both copies of this SNP had reduced TLR1 expression on the cell surface and abrogated downstream signaling to soluble TLR1/2 agonists (25, 26), whereas total intracellular levels of TLR1 were unaffected (45). The decreased downstream signaling resulted in dampened immune responses to mycobacterial infection (25) which had a protective role in tuberculosis and leprosy (13). Conversely, in response to *B. burgdorferi*, 1805GG leads to enhanced inflammatory responses (17). This variability in response between *B. burgdorferi* and other pathogens likely reflects the differences in microbial antigens that engage TLR1/2 or other host pathogen sensing receptors.

A central question then is how does variation in pathogen-sensing genes such as TLR1 contribute to persistent inflammation and dysregulated immune responses for months to years after antibiotic therapy? One possibility is that retained spirochetal antigens, such as *B. burgdorferi* peptidoglycans, continue to trigger immune activation through intracellular NOD-like receptors (46–49). Genes encoding several PRRs (including *TLR2*, *TLR5*, and *NOD2*) are upregulated in response to *B. burgdorferi* stimulation in TLR1-1805GG cells, perhaps to compensate for diminished TLR1 signaling on the cell surface and may contribute to the overall inflammatory response. In addition, impaired TLR1 signaling appears to result in lack of appropriate immune regulation. This is exemplified by a break in innate immune tolerance to repeat stimulation with *B. burgdorferi* and a concomitant upregulation of immune genes as observed in PBMCs with this TLR1-1805G SNP. Several of the induced genes, particularly proinflammatory cytokines *IL1β, IL6*, and *PTGS2* and genes involved in antigen presentation (*HLA-DRB1*), have been implicated in persistent inflammation in other conditions including atopic dermatitis, psoriasis, and rheumatoid arthritis (50, 51).

This break in tolerance is characterized by sustained elevated protein levels of cytokines and chemokines associated with both innate (IFNα, IL-1β, IL-6, TNF) and adaptive Th1 (IFNγ, CXCL9) or Th17 (IL-17A, GM-CSF) mediators in cell supernatants and in patients (14, 15, 17, 19, 35, 52). The heightened inflammation observed in PBMCs with the TLR1 SNP was also shown in THP-1 macrophages lacking the TLR1 receptor. However, TLR1-KO THP-1 cells produced markedly lower levels of IL-6 following primary stimulation with *B. burgdorferi* compared to the robust cytokine induction in TLR1-1805GG PBMCs. This difference likely reflects the complete absence of TLR1 in knockout cells, whereas in PBMCs with the SNP, TLR1 is mislocalized to intracellular compartments where it may contribute to aberrant signaling (26). Despite the differences in IL-6 induction between THP-1 KOs and PBMCs with TLR1 SNP, the net result of TLR1 deficiency on the cell surface with both cell models is a heightened and sustained pro-inflammatory response.

These *in vitro* findings are reminiscent of inflammatory responses in patients with post-infectious LA in whom high levels of some of the same mediators found in PBMCs (IL-6, TNF, IFNγ, CXCL9, and IL-17) are thought to contribute to dysregulated immune responses and pathology in joints (15, 19, 35). The levels of CXCL9, an IFNγ-inducible chemokine, can exceed 250,000 pg/ml in synovial fluid of post-infectious LA patients with TLR1-1805GG and are associated with recruitment of CD4+ T effector cells, some of which may be autoreactive (14). Similarly, high levels of Th17 mediators, including IL-23 and IL-17, correlate directly with autoantibody responses and are implicated in joint pathology. In contrast, patients with antibiotic-responsive LA appear to downregulate these inflammatory immune responses and their arthritis resolves (14). Thus, in our *in vitro* studies, as in patients, the excessive inflammatory response coincides with a lack of appropriate immune regulation. This exacerbated inflammation, which appears to be decoupled from the infection, requires specific treatment. After antibiotics, patients with post-infectious LA are usually treated successfully with Disease-Modifying Antirheumatic Drugs (DMARDs) as is the standard of care for other prototypical chronic inflammatory arthritides (29).

This study has several limitations. Although the findings presented here are based on the largest cohort of patients with LA, this sample size is still relatively small for genetic association studies. Nevertheless, the current study validates a greater frequency of TLR1-1805GG genotype in patients with post-infectious LA reported previously (17), and we expand the comparison to European patients with EM and to larger cohorts in the general population. Another possible limitation is that although PBMC donors did not have a known history of Lyme disease, prior exposure to Borrelia could not be fully excluded, and could potentially influence immune responses in this study. However, the data across PBMCs from multiple healthy donors in the current study as well as previous results in PBMCs from LA patients (17) and in THP-1 cells lacking TLR1 demonstrate greater inflammatory responses to *B. burgdorferi* in cells with TLR1 deficiency. In addition, the RNA-seq analysis was performed using PBMCs from a single TLR1-1805TT and a single TLR1-1805GG donor, which limits generalizability of the results. However, the transcriptional patterns observed in these donors, including the sustained upregulation of cytokines (such as *IL6*) and other immune genes, are consistent with results in multiple donors, and the overall greater inflammatory phenotype in patients with TLR1-1805GG SNP. Ultimately, elucidating the wide range of pathways that mediate these responses will require a more comprehensive and in-depth analysis. Finally, TLR1-1805G SNP is likely one of many host risk factors associated with severe Lyme disease. For example, we recently discovered that several SNPs in Late Cornified Envelope 3 (LCE3) genes contribute to greater Th17 inflammatory responses and are risk factors for post-infectious LA (20). Thus, SNPs in multiple loci may predispose patients to severe disease. Moreover, SNPs in other genes have recently been implicated in susceptibility to Lyme disease (23, 53). These initial studies highlight the important role of host genetic variability in shaping immune responses and provide a new paradigm for comprehensive genomic interrogations in patients with various clinical presentations of Lyme disease.

Collectively our findings demonstrate that severe disease in patients with the TLR1-1805GG genotype may be due to a failure to induce immune tolerance, resulting in unabated and sustained inflammation. These results offer a potential explanation for how host genetics impact initial events in the *Borrelia* infection and set the stage for dysregulated immune responses months to years after treatment and apparent resolution of the infection. Furthermore, the results suggest that functional TLR1 signaling is critically important in modulating the amplitude and duration of immune responses to microbial stimuli.

## Data Availability

Original datasets are available in a publicly accessible repository: The original contributions presented in the study are publicly available. This data can be found here: [https://www.ebi.ac.uk/biostudies/arrayexpress/E-MTAB-16016].

## References

[1] Stanek G Wormser GP Gray J Strle F . Lyme borreliosis. Lancet. (2012) 379:461–73. doi: 10.1016/S0140-6736(11)60103-7, PMID: 21903253

[2] Steere AC Strle F Wormser GP Hu LT Branda JA Hovius JW . Lyme borreliosis. Nat Rev Dis Primers. (2016) 2:16090. doi: 10.1038/nrdp.2016.90, PMID: 27976670 PMC5539539

[3] Steere AC Angelis SM . Therapy for Lyme arthritis: strategies for the treatment of antibiotic-refractory arthritis. Arthritis Rheum. (2006) 54:3079–86. doi: 10.1002/art.22131, PMID: 17009226

[4] Lochhead RB Strle K Arvikar SL Weis JJ Steere AC . Lyme arthritis: linking infection, inflammation and autoimmunity. Nat Rev Rheumatol. (2021) 17:449–61. doi: 10.1038/s41584-021-00648-5, PMID: 34226730 PMC9488587

[5] Li X McHugh GA Damle N Sikand VK Glickstein L Steere AC . Burden and viability of Borrelia burgdorferi in skin and joints of patients with erythema migrans or lyme arthritis. Arthritis Rheumatism. (2011) 63:2238–47. doi: 10.1002/art.30384, PMID: 21590753 PMC5444298

[6] Barbour AG Jasinskas A Kayala MA Davies DH Steere AC Baldi P . A genome-wide proteome array reveals a limited set of immunogens in natural infections of humans and white-footed mice with Borrelia burgdorferi. Infect Immun. (2008) 76:3374–89. doi: 10.1128/IAI.00048-08, PMID: 18474646 PMC2493225

[7] Gorvel J-P Oosting M ter Hofstede H Sturm P Adema GJ Kullberg B-J . TLR1/TLR2 Heterodimers Play an Important Role in the Recognition of Borrelia Spirochetes. PLoS One. (2011) 6(10):e25998. doi: 10.1371/journal.pone.0025998, PMID: 21998742 PMC3187844

[8] Singh SK Girschick HJ . Toll-like receptors in Borrelia burgdorferi-induced inflammation. Clin Microbiol Infect. (2006) 12:705–17. doi: 10.1111/j.1469-0691.2006.01440.x, PMID: 16842565

[9] Cervantes JL La Vake CJ Weinerman B Luu S O'Connell C Verardi PH . Human TLR8 is activated upon recognition of Borrelia burgdorferi RNA in the phagosome of human monocytes. J Leukoc Biol. (2013) 94:1231–41. doi: 10.1189/jlb.0413206, PMID: 23906644 PMC3828603

[10] Petzke MM Brooks A Krupna MA Mordue D Schwartz I . Recognition of Borrelia burgdorferi, the Lyme disease spirochete, by TLR7 and TLR9 induces a type I IFN response by human immune cells. J Immunol. (2009) 183:5279–92. doi: 10.4049/jimmunol.0901390, PMID: 19794067

[11] Cervantes JL Dunham-Ems SM La Vake CJ Petzke MM Sahay B Sellati TJ . Phagosomal signaling by Borrelia burgdorferi in human monocytes involves Toll-like receptor (TLR) 2 and TLR8 cooperativity and TLR8-mediated induction of IFN-beta. Proc Natl Acad Sci U S A. (2011) 108:3683–8. doi: 10.1073/pnas.1013776108, PMID: 21321205 PMC3048123

[12] Hart BE Tapping RI . Cell surface trafficking of TLR1 is differentially regulated by the chaperones PRAT4A and PRAT4B. J Biol Chem. (2012) 287:16550–62. doi: 10.1074/jbc.M112.342717, PMID: 22447933 PMC3351281

[13] Hart BE Tapping RI . Genetic Diversity of Toll-Like Receptors and Immunity to M. leprae infection. J Trop Med. (2012) 2012:415057. doi: 10.1155/2012/415057, PMID: 22529866 PMC3317006

[14] Strle K Sulka KB Pianta A Crowley JT Arvikar SL Anselmo A . T-Helper 17 Cell Cytokine Responses in Lyme Disease Correlate With Borrelia burgdorferi Antibodies During Early Infection and With Autoantibodies Late in the Illness in Patients With Antibiotic-Refractory Lyme Arthritis. Clin Infect Dis. (2017) 64:930–8. doi: 10.1093/cid/cix002, PMID: 28077518 PMC5850331

[15] Strle K Stupica D Drouin EE Steere AC Strle F . Elevated levels of IL-23 in a subset of patients with post-lyme disease symptoms following erythema migrans. Clin Infect Dis. (2014) 58:372–80. doi: 10.1093/cid/cit735, PMID: 24218102 PMC3890340

[16] Akira S Takeda K . Toll-like receptor signalling. Nat Rev Immunol. (2004) 4:499–511. doi: 10.1038/nri1391, PMID: 15229469

[17] Strle K Shin JJ Glickstein LJ Steere AC . Association of a Toll-like receptor 1 polymorphism with heightened Th1 inflammatory responses and antibiotic-refractory Lyme arthritis. Arthritis Rheum. (2012) 64:1497–507. doi: 10.1002/art.34383, PMID: 22246581 PMC3338893

[18] Shin JJ Strle K Glickstein LJ Luster AD Steere AC . Borrelia burgdorferi stimulation of chemokine secretion by cells of monocyte lineage in patients with Lyme arthritis. Arthritis Res Ther. (2010) 12:R168. doi: 10.1186/ar3128, PMID: 20828409 PMC2990995

[19] Ehrbar D Arvikar SL Sulka KB Chiumento G Nelson NLJ Hernandez SA . Variants in the Late Cornified Envelope Gene Locus Are Associated With Elevated T-helper 17 Responses in Patients With Postinfectious Lyme Arthritis. J Infect Dis. (2024) 230:S40–50. doi: 10.1093/infdis/jiae164, PMID: 39140723 PMC11322884

[20] Botey-Bataller J Vrijmoeth HD Ursinus J Kullberg BJ van den Wijngaard CC ter Hofstede H . A comprehensive genetic map of cytokine responses in Lyme borreliosis. Nat Commun. (2024) 15:3795. doi: 10.1038/s41467-024-47505-z, PMID: 38714679 PMC11076587

[21] Bramwell KK Ma Y Weis JH Chen X Zachary JF Teuscher C . Lysosomal beta-glucuronidase regulates Lyme and rheumatoid arthritis severity. J Clin Invest. (2014) 124:311–20. doi: 10.1172/JCI72339, PMID: 24334460 PMC3871255

[22] Bramwell KK Mock K Ma Y Weis JH Teuscher C Weis JJ . beta-Glucuronidase, a Regulator of Lyme Arthritis Severity, Modulates Lysosomal Trafficking and MMP-9 Secretion in Response to Inflammatory Stimuli. J Immunol. (2015) 195:1647–56. doi: 10.4049/jimmunol.1500212, PMID: 26170381 PMC4530054

[23] Strausz S Abner E Blacker G Galloway S Hansen P Feng Q . SCGB1D2 inhibits growth of Borrelia burgdorferi and affects susceptibility to Lyme disease. Nat Commun. (2024) 15:2041. doi: 10.1038/s41467-024-45983-9, PMID: 38503741 PMC10950847

[24] Ogrinc K Hernandez SA Korva M Bogovic P Rojko T Lusa L . Unique Clinical, Immune, and Genetic Signature in Patients with Borrelial Meningoradiculoneuritis. Emerg Infect Dis. (2022) 28:766–76. doi: 10.3201/eid2804.211831, PMID: 35318928 PMC8962912

[25] Hawn TR Misch EA Dunstan SJ Thwaites GE Lan NT Quy HT . A common human TLR1 polymorphism regulates the innate immune response to lipopeptides. Eur J Immunol. (2007) 37:2280–9. doi: 10.1002/eji.200737034, PMID: 17595679

[26] Hart BE Tapping RI . Differential trafficking of TLR1 I602S underlies host protection against pathogenic mycobacteria. J Immunol. (2012) 189:5347–55. doi: 10.4049/jimmunol.1201545, PMID: 23105135 PMC3504178

[27] Sellati TJ Sahay B Wormser GP . The Toll of a TLR1 polymorphism in lyme disease: a tale of mice and men. Arthritis Rheum. (2012) 64:1311–5. doi: 10.1002/art.34386, PMID: 22246662

[28] Steere AC Glickstein L . Elucidation of Lyme arthritis. Nat Rev Immunol. (2004) 4:143–52. doi: 10.1038/nri1267, PMID: 15040587

[29] Arvikar SL Steere AC . Diagnosis and treatment of Lyme arthritis. Infect Dis Clin North Am. (2015) 29:269–80. doi: 10.1016/j.idc.2015.02.004, PMID: 25999223 PMC4443866

[30] Genomes Project C Auton A Brooks LD Durbin RM Garrison EP Kang HM . A global reference for human genetic variation. Nature. (2015) 526:68–74. doi: 10.1038/nature15393, PMID: 26432245 PMC4750478

[31] Strle K Jones KL Drouin EE Li X Steere AC . Borrelia burgdorferi RST1 (OspC type A) genotype is associated with greater inflammation and more severe Lyme disease. Am J Pathol. (2011) 178:2726–39. doi: 10.1016/j.ajpath.2011.02.018, PMID: 21641395 PMC3123987

[32] Strle K Drouin EE Shen S El Khoury J McHugh G Ruzic-Sabljic E . Borrelia burgdorferi stimulates macrophages to secrete higher levels of cytokines and chemokines than Borrelia afzelii or Borrelia garinii. J Infect Dis. (2009) 200:1936–43. doi: 10.1086/648091, PMID: 19909078 PMC2783242

[33] Oosting M Brouwer M Vrijmoeth HD Pascual Domingo R Greco A ter Hofstede H . Borrelia burgdorferi is strong inducer of IFN-gamma production by human primary NK cells. Cytokine. (2022) 155:155895. doi: 10.1016/j.cyto.2022.155895, PMID: 35569383

[34] Salazar JC Duhnam-Ems S La Vake C Cruz AR Moore MW Caimano MJ . Activation of human monocytes by live Borrelia burgdorferi generates TLR2-dependent and -independent responses which include induction of IFN-beta. PLoS Pathog. (2009) 5:e1000444. doi: 10.1371/journal.ppat.1000444, PMID: 19461888 PMC2679197

[35] Shin JJ Glickstein LJ Steere AC . High levels of inflammatory chemokines and cytokines in joint fluid and synovial tissue throughout the course of antibiotic-refractory lyme arthritis. Arthritis Rheum. (2007) 56:1325–35. doi: 10.1002/art.22441, PMID: 17393419

[36] Sahay B Patsey RL Eggers CH Salazar JC Radolf JD Sellati TJ . CD14 signaling restrains chronic inflammation through induction of p38-MAPK/SOCS-dependent tolerance. PLoS Pathog. (2009) 5:e1000687. doi: 10.1371/journal.ppat.1000687, PMID: 20011115 PMC2781632

[37] Foster SL Hargreaves DC Medzhitov R . Gene-specific control of inflammation by TLR-induced chromatin modifications. Nature. (2007) 447:972–8. doi: 10.1038/nature05836, PMID: 17538624

[38] Biswas SK Lopez-Collazo E . Endotoxin tolerance: new mechanisms, molecules and clinical significance. Trends Immunol. (2009) 30:475–87. doi: 10.1016/j.it.2009.07.009, PMID: 19781994

[39] Zhou Y Zhou B Pache L Chang M Khodabakhshi AH Tanaseichuk O . Metascape provides a biologist-oriented resource for the analysis of systems-level datasets. Nat Commun. (2019) 10:1523. doi: 10.1038/s41467-019-09234-6, PMID: 30944313 PMC6447622

[40] Akhmedov M Martinelli A Geiger R Kwee I . Omics Playground: a comprehensive self-service platform for visualization, analytics and exploration of Big Omics Data. NAR Genom Bioinform. (2020) 2:lqz019. doi: 10.1093/nargab/lqz019, PMID: 33575569 PMC7671354

[41] Tanaka T Narazaki M Kishimoto T . IL-6 in inflammation, immunity, and disease. Cold Spring Harb Perspect Biol. (2014) 6:a016295. doi: 10.1101/cshperspect.a016295, PMID: 25190079 PMC4176007

[42] Shin E-C Soloski MJ Crowder LA Lahey LJ Wagner CA Robinson WH . Serum Inflammatory Mediators as Markers of Human Lyme Disease Activity. PLoS One. (2014) 9(4):e93243. doi: 10.1371/journal.pone.0093243, PMID: 24740099 PMC3989169

[43] Naidoo L Arumugam T Ramsuran V . Host Genetic Impact on Infectious Diseases among Different Ethnic Groups. Adv Genet (Hoboken). (2023) 4:2300181. doi: 10.1002/ggn2.202300181, PMID: 38099246 PMC10716055

[44] Deschamps M Laval G Fagny M Itan Y Abel L Casanova JL . Genomic Signatures of Selective Pressures and Introgression from Archaic Hominins at Human Innate Immunity Genes. Am J Hum Genet. (2016) 98:5–21. doi: 10.1016/j.ajhg.2015.11.014, PMID: 26748513 PMC4716683

[45] Johnson CM Lyle EA Omueti KO Stepensky VA Yegin O Alpsoy E . Cutting edge: A common polymorphism impairs cell surface trafficking and functional responses of TLR1 but protects against leprosy. J Immunol. (2007) 178:7520–4. doi: 10.4049/jimmunol.178.12.7520, PMID: 17548585

[46] Jutras BL Lochhead RB Kloos ZA Biboy J Strle K Booth CJ . Borrelia burgdorferi peptidoglycan is a persistent antigen in patients with Lyme arthritis. Proc Natl Acad Sci U S A. (2019) 116:13498–507. doi: 10.1073/pnas.1904170116, PMID: 31209025 PMC6613144

[47] Berende A Oosting M Kullberg BJ Netea MG Joosten LA . Activation of innate host defense mechanisms by Borrelia. Eur Cytokine Netw. (2010) 21:7–18. doi: 10.1684/ecn.2009.0179, PMID: 20146985

[48] Petnicki-Ocwieja T DeFrancesco AS Chung E Darcy CT Bronson RT Kobayashi KS . Nod2 suppresses Borrelia burgdorferi mediated murine Lyme arthritis and carditis through the induction of tolerance. PLoS One. (2011) 6:e17414. doi: 10.1371/journal.pone.0017414, PMID: 21387014 PMC3046161

[49] Petnicki-Ocwieja T Kern A . Mechanisms of Borrelia burgdorferi internalization and intracellular innate immune signaling. Front Cell Infect Microbiol. (2014) 4:175. doi: 10.3389/fcimb.2014.00175, PMID: 25566512 PMC4266086

[50] Kragstrup TW Otkjaer K Holm C Jorgensen A Hokland M Iversen L . The expression of IL-20 and IL-24 and their shared receptors are increased in rheumatoid arthritis and sp*ondyloarthropathy*. Cytokine. (2008) 41:16–23. doi: 10.1016/j.cyto.2007.10.004, PMID: 18061474

[51] Zhong Y Zhang X Chong W . Interleukin-24 Immunobiology and Its Roles in Inflammatory Diseases. Int J Mol Sci. (2022) 23:627. doi: 10.3390/ijms23020627, PMID: 35054813 PMC8776082

[52] Hernandez SA Ogrinc K Korva M Kastrin A Bogovic P Rojko T . Association of Persistent Symptoms after Lyme Neuroborreliosis and Increased Levels of Interferon-alpha in Blood. Emerg Infect Dis. (2023) 29:1091–101. doi: 10.3201/eid2906.221685, PMID: 37209716 PMC10202885

[53] Vrijmoeth HD Ursinus J Botey-Bataller J Kuijpers Y Chu X van de Schoor FR . Genome-wide analyses in Lyme borreliosis: identification of a genetic variant associated with disease susceptibility and its immunological implications. BMC Infect Dis. (2024) 24:337. doi: 10.1186/s12879-024-09217-z, PMID: 38515037 PMC10956190

