# Supplementary material for: Toll-like receptor 1 polymorphism is associated with impaired immune tolerance, dysregulated inflammatory responses to Borrelia burgdorferi, and heightened risk of post-infectious Lyme arthritis
Source: Front Immunol. 2025 Nov 17;16:1711765. doi: 10.3389/fimmu.2025.1711765 (PMC12665665; doi:10.3389/fimmu.2025.1711765)
Supplement: Supplementary file 1 [file DataSheet1.pdf]

Supplementary Table 1: TLR1-1805 genetic modeling indicates an association between clinical outcomes of Lyme arthritis and a G-allele recessive model.

**Model**	**Antibiotic-responsive LA *n* (%)**	**Post-infectious LA *n* (%)**	**OR**	**95% CI**	***p*-Value**	**AIC**
Recessive						
T/T–T/G	65 (57)	68 (40)	1.0	–	0.004	379.4
G/G	49 (43)	103 (60)	2.01	1.24 to 3.25		
Log-additive*						
0, 1, 2	114 (40)	171 (60)	1.62	1.11 to 2.36	0.01	381.0
Codominant						
T/T (reference)	11 (10)	12 (7)	1.0	–	0.02	381.4
T/G	54 (47)	56 (33)	0.95	0.39 to 2.33		
G/G	49 (43)	103 (60)	1.92	0.80 to 4.67		
Overdominant						
T/T–G/G	60 (53)	115 (67)	1.0	–	0.01	381.5
T/G	54 (47)	56 (33)	0.54	0.33 to 0.88		
Dominant						
T/T	11 (10)	12 (7)	1.0	–	0.4	386.9
T/G–G/G	103 (90)	159 (93)	1.42	0.60 to 3.33		

* Entries are group totals for this model, OR is per G-allele

Abbreviations: TLR, toll-like receptor; LA, Lyme arthritis; CI, confidence interval; OR, odds ratio; AIC, Akaike Information Criterion

Recessive model = best fit (lowest AIC score) and indicates G/G increases odds of post-infectious LA. Log-additive suggests that odds of post-infectious LA increase by ~62% for each additional G allele. Overdominant OR <1 suggests heterozygote genotype T/G is less frequent among post-infectious LA patients which is compatible with a recessive risk allele.
